# Cell-to-Cell Communications in Alcohol-Associated Liver Disease

**DOI:** 10.3389/fphys.2022.831004

**Published:** 2022-02-21

**Authors:** Natalia A. Osna, Akiko Eguchi, Ariel E. Feldstein, Hidekazu Tsukamoto, Raghubendra S. Dagur, Murali Ganesan, Moses New-Aaron, Madan Kumar Arumugam, Srinivas Chava, Marcelle Ribeiro, Gyongyi Szabo, Sebastian Mueller, Shijin Wang, Cheng Chen, Steven A. Weinman, Kusum K. Kharbanda

**Affiliations:** ^1^Research Service, Veterans Affairs Nebraska-Western Iowa Health Care System, Omaha, NE, United States; ^2^Department of Internal Medicine, University of Nebraska Medical Center, Omaha, NE, United States; ^3^Department of Gastroenterology and Hepatology, Graduate School of Medicine, Mie University, Tsu, Japan; ^4^Department of Pediatrics, University of California, San Diego, San Diego, CA, United States; ^5^Southern California Research Center for ALPD and Cirrhosis and Department of Pathology, Keck School of Medicine of the University of Southern California, Los Angeles, CA, United States; ^6^Greater Los Angeles VA HealthCare System, Los Angeles, CA, United States; ^7^Department of Environmental Health, Occupational Health, and Toxicology, College of Public Health, University of Nebraska Medical Center, Omaha, NE, United States; ^8^Harvard Medical School and Department of Medicine, Beth Israel Deaconess Medical Center, Boston, MA, United States; ^9^Salem Medical Center and Center for Alcohol Research, University of Heidelberg, Heidelberg, Germany; ^10^Department of Internal Medicine, University of Kansas Medical Center, Kansas City, KS, United States; ^11^Department of Biochemistry and Molecular Biology, University of Nebraska Medical Center, Omaha, NE, United States

**Keywords:** alcohol hepatitis, extracellular vesicles, pyroptosis, liver stiffness, HIV, fibrosis

## Abstract

This review covers some important new aspects of the alcohol-induced communications between liver parenchymal and non-parenchymal cells leading to liver injury development. The information exchange between various cell types may promote end-stage liver disease progression and involves multiple mechanisms, such as direct cell-to-cell interactions, extracellular vesicles (EVs) or chemokines, cytokines, and growth factors contained in extracellular fluids/cell culture supernatants. Here, we highlighted the role of EVs derived from alcohol-exposed hepatocytes (HCs) in activation of non-parenchymal cells, liver macrophages (LM), and hepatic stellate cells (HSC). The review also concentrates on EV-mediated crosstalk between liver parenchymal and non-parenchymal cells in the settings of HIV- and alcohol co-exposure. In addition, we overviewed the literature on the crosstalk between cell death pathways and inflammasome activation in alcohol-activated HCs and macrophages. Furthermore, we covered highly clinically relevant studies on the role of non-inflammatory factors, sinusoidal pressure (SP), and hepatic arterialization in alcohol-induced hepatic fibrogenesis. We strongly believe that the review will disclose major mechanisms of cell-to-cell communications pertained to alcohol-induced liver injury progression and will identify therapeutically important targets, which can be used for alcohol-associated liver disease (ALD) prevention.

## Introduction

Hepatocytes (HCs), the parenchymal cells of the liver, comprise ~70% of the liver mass, while the non-parenchymal cells, including Kupffer cells (KCs), liver sinusoidal endothelial cells (LSECs), hepatic stellate cells (HSCs), and liver-associated lymphocytes make up the remaining 15–30%. Each liver cell type plays a specific role, not only in normal hepatic physiology, but also in initiating and perpetuating liver injury.

The crosstalk between liver HCs and non-parenchymal cells can occur *via* direct cell-to-cell interactions, ligand-receptor interactions, cytokines/chemokines, hormones, and extracellular vesicles (EVs) including exosomes, microvesicles, and apoptotic bodies. While this crosstalk operates to maintain liver homeostasis, it also plays a crucial role in disease development, initiated or exacerbated by communications between parenchymal and non-parenchymal liver cells.

**Figure 1:**
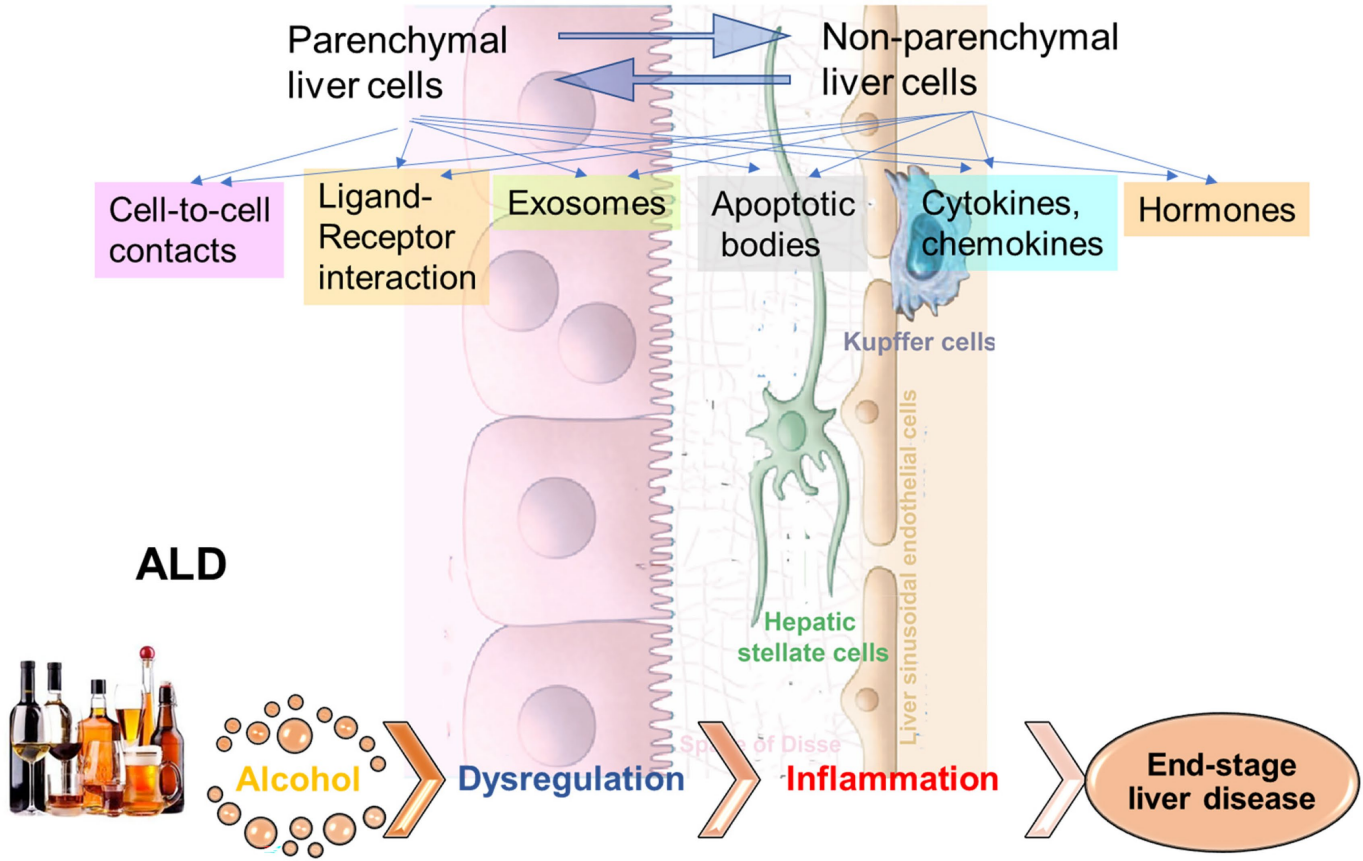
Cell-to-cell communications in ethanol-related progression to end-stage liver diseases. The signals to activate the crosstalk between liver parenchymal and non-parenchymal cells can come *via* direct cell-to-cell interactions, *via* ligand-receptor interactions, exosomes, apoptotic bodies, cytokines/chemokines, hormones, etc. Exposure to alcohol, especially, ethanol metabolites induce activation of signal transduction pathways in recipient cells, thereby altering their functions, which leads to liver inflammation and fibrosis development, with progression to end-stage liver disease.

Alcohol-associated liver disease (ALD) represents a spectrum of liver injury resulting from alcohol use, ranging from hepatic steatosis to more advanced forms including alcohol-associated steatohepatitis (ASH), alcohol-associated cirrhosis (AC), and acute alcoholic hepatitis (AH) presenting as acute-on-chronic liver failure (Crabb et al., 2020). ALD is a major cause of liver disease worldwide, both on its own and as a co-factor in the progression of chronic viral hepatitis, and other liver diseases (Crabb et al., 2020). Exposure to alcohol, and especially ethanol metabolites, affects activation of signal transduction pathways in recipient cells, thereby altering their functions leading to liver inflammation and fibrosis development (Figure 1). In addition, “biomechanical” factors like sinusoidal pressure (SP) and HSC stretch may further amplify cell-to-cell communications leading to liver fibrosis onset contributing to cirrhosis and progression to end-stage liver disease.

Cell-to-cell communications is very broad and multifactorial topic, and all aspects attributed to ALD cannot be overviewed in the frame of current article. Here, we review the contribution of selected, but crucial for end-stage liver disease pathogenesis factors analyzed in the context of alcohol exposure. These factors may regulate liver cell-to-cell communications separately or promote the crosstalk between liver cells in an interdependent manner.

## EVs as Key Mediators of HSC and Macrophage Activation in ALD

Chronic ethanol consumption results in lipid accumulation in HCs and organelle stress leading to liver inflammation and fibrosis in the progression of ALD. The crosstalk between parenchymal cells and non-parenchymal cells including liver macrophages (LM) and HSC is crucial to this process. However, the molecular mechanisms and signaling pathways involved in the crosstalk between lipid overloaded HC and non-parenchymal cells, remain poorly understood. EVs are released from damaged/stressed cells with a cargo that may provide a barcode of the cell of origin, such as mRNAs, non-coding RNAs, selective microRNAs (miRNAs), proteins, and mtDNAs. EVs includes mainly three different types of vesicles, which are exosomes, microvesicles, and apoptotic body (Yanez-Mo et al., 2015). The characterization of these vesicles is as follow; exosomes have 30–100 nm size and produced by intraluminal vesicles within multivesicular bodies. Microvesicles have 100–1,000 nm size and produced by the budding of plasma membrane. Apoptotic body has 1,000–5,000 nm size and produced by the budding of plasma membrane during apoptosis. Although various markers are identified for each of vesicles, some of the markers including CD9 are expressed in both, exosomes and microvesicles (Yanez-Mo et al., 2015). In addition, the size cannot identify the difference microvesicles and exosomes (Tkach and Thery, 2016). As for EV biological function, EVs are indispensable for cell-to-cell communicators and can be detected in circulation (Kalluri and LeBleu, 2020). EVs are taken up into target cells and modulate cell signaling and there is growing recognition that they serve a key role in cell-to-cell communication (Eguchi et al., 2019). In response to various stresses or pathological stimuli, EVs are released by various types of cells, such as HCs, macrophages (Kupffer cells and infiltrating macrophages), and others and these EVs reflect the disease state and cell of origin in terms of numbers and cargo specificity (Verma et al., 2016; Dagur et al., 2020). Indeed, HC and non-parenchymal cells release EVs and activate targets cells involved in the progression of liver diseases such as nonalcoholic steatohepatitis and ALD (Eguchi et al., 2019; Thietart and Rautou, 2020). However, the information about EV contributions to liver disease progression triggered by ethanol is very limited.

**Figure 2:**
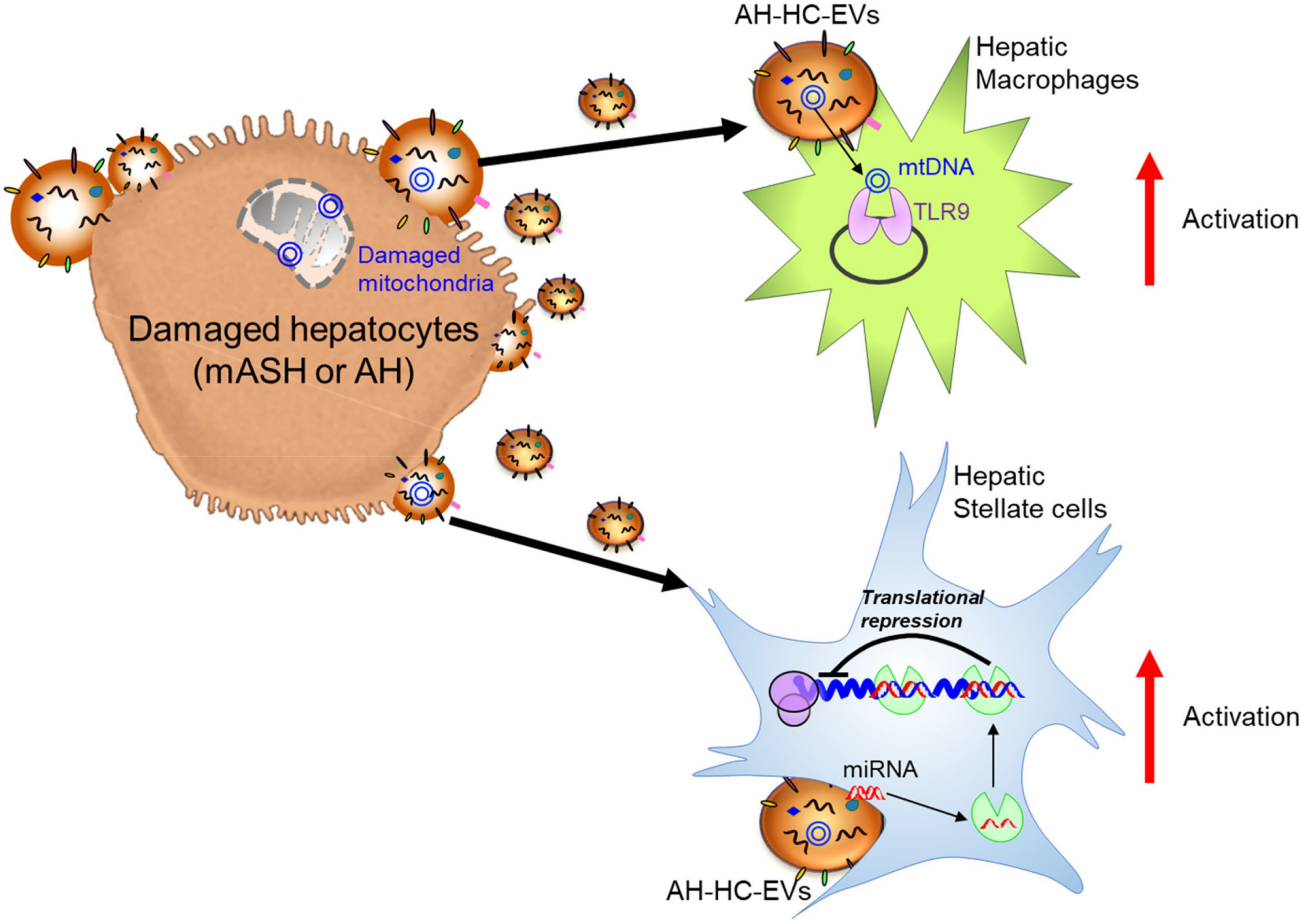
Damaged hepatocyte released extracellular vesicles (EVs) and hepatocyte-derived EVs (HC-EVs) activate target cells with EV composition. Alcoholic hepatitis (AH)-HC-EVs may contribute the progression of diseases in multiple pathways; microRNAs (miRNAs) activate hepatic stellate cells through suppression of target genes and mitochondrial (mt)DNAs activate hepatic macrophages through toll-like receptor 9 (TLR9).

In ALD, a significant amount of HC-EVs, which contained CD40 ligand (CD40L), was found to be released in a caspase-3-dependent-manner from HepG2 cells treated with EtOH and overexpressing cytochrome P450 2E1, an enzyme involved in ethanol metabolism (Verma et al., 2016). CD40L-containing HC-EVs activated macrophages switch to the M1 type inflammatory phenotype. In a chronic ethanol fed Lieber-DeCarli diet plus single binge ethanol feeding model (NIAAA model), wild-type mice with a genetic deletion of either CD40 (CD40−/−) or the caspase-activating tumor necrosis factor-related apoptosis-inducing ligand (TRAIL) receptor (TR−/−), were protected from ethanol-induced liver injury due to the attenuation of macrophage infiltration. In addition, CD40L containing circulating EVs were elevated in AH patients. In the NIAAA model, the increase in HC-EVs encapsulating mtDNA was associated with the activation of hepatic ER stress, as well as inflammasome activation, and this induced neutrophilic inflammation through TLR9 activation, thereby resulting in the acceleration of HC damage in a feed-forward loop (Cai et al., 2017). The magnitude of neutrophil infiltration in the liver was attenuated in transcription factor C/EBP homologous protein (Chop) KO mice, Jun-amino-terminal kinase 2 (JNK2) KO mice, or caspase-1 inhibitor-treated mice. In Lieber-DeCarli diet mice, which results in mild changes in liver pathology compared to the NIAAA model, transferred circulating EVs (including HC-EVs) into naïve mice demonstrated an increase of MCP-1 mRNA levels in HCs and elevated inflammatory M1 type KC and infiltrating monocytes (Saha et al., 2018). The activation of cultured mouse macrophages, RAW cells, was induced by heat shock protein 90 (Hsp90) enriched in circulating EVs. Cell-to-cell communication by HC-EVs was also observed using ethanol intragastric infusion models, such as mild alcoholic steatohepatitis (mASH) established by 4 weeks of ethanol intragastric infusion resulting in steatosis and mild mononuclear cell inflammation (Ueno et al., 2012) and acute on chronic AH established by superimposing weekly alcohol binge administration to intragastric ethanol-infused mice, which mimics human AH including hepatocellular damage with ballooning cell degeneration, PMN inflammation, fibrosis, and ductular reaction (Lazaro et al., 2015). HC-EVs were directly purified from the supernatant of culture medium using isolated mASH or AH HC-EVs. Treatment of LMs isolated from wild-type mice with mASH-HC-EVs resulted in a significant upregulation of various macrophage activation markers such as interleukin (IL)-1β, IL-6, and tumor necrosis factor-α (TNFα). In HSCs isolated from AH mice with liver fibrosis, AH-HC-EVs induced α-smooth muscle actin (SMA) and collagen type 1 alpha 1 chain (Col1a1; Eguchi et al., 2020). Further, 149 of 1,020 genes including nuclear receptor subfamily 1 group D member 2 (Nr1d2), a quiescent HSC marker and SMAD family member 7 (Smad7), a negative regulator of HSC activation was differentially regulated in HSC from AH mice vs. controls and served as the predicted targets of 20 miRNAs upregulated in AH-HC-EVs. The Nr1d2 gene, which was downregulated in HSCs from the AH mice, served as the predicted target of miR-27a and miR-181, which belong to 20 miRNAs upregulated in AH-HC-EVs. Indeed, transfection of miR-27a and -181 in HSCs led to a reduction of Nr1d2 expression. Additionally, AH-HC-EVs were enriched with mitochondrial DNA (mtDNAs) and upregulated IL-1β and IL-17 production by LMs from AH mice in a TLR9-dependent manner. These results demonstrated that AH-HC-EVs modulated liver fibrogenesis by directly targeting the quiescent HSC transcripts *via* miRNAs and by amplifying HSC activation *via* mtDNAs-triggered induction of profibrogenic IL-1β and IL-17 by LMs (Eguchi et al., 2020). These studies reveal that when HCs are damaged by alcohol, they are a key source of EVs that impact the subsequent course of disease. However, the exact nature of these EVs, as well as the key target cells differs depending on the severity of the alcohol injury. In mild ASH (mASH), EVs prominently trigger inflammatory responses in macrophages (Figure 2). In more severe AH, the EVs contain a mixture of miRNAs that induce stellate cell activation *via* translational repression leading to fibrosis; they also contain mtDNAs to induce macrophage activation *via* TLR9 response (Figure 2).

Non-parenchymal cell-derived EVs also contribute to the progression of ALD. Monocytes/macrophages release miR-27a enriched EVs by EtOH in a dose dependent-manner, and these EVs mediated differentiation of naïve monocytes to M2 macrophages (Saha et al., 2016). These findings uncover EVs as potential novel therapeutic targets for ALD.

## Viral Infections Potentiate ALD Pathogenesis

Alcohol serves as a powerful trigger of EV release, which establishes a cross-talk between parenchymal and non-parenchymal liver cells. Hepatotropic viruses further promote EV production in alcohol-pre-sensitized cells and affect EVs cargo. Unfortunately, very few studies address dual effects of viral infections and ethanol metabolism on liver cell-to-cell communications *via* EVs.

In HBV-infection, EVs released from HBV-infected hepatocytes contained viral nucleic acids and induced NKG2D ligand expression in macrophages by stimulating MyD88, TICAM-1, and MAVS-dependent pathways. In addition, depletion of exosomes from EVs markedly reduced NKG2D ligand expression, suggesting the importance of exosomes for NK cell activation (Kouwaki et al., 2016). This study was not performed in the context of alcohol. However, exosomes contain HBV RNA and HBV DNA (Yang et al., 2017). While the expression of virus goes up in hepatocytes exposed to ethanol (Ganesan et al., 2019a, 2021), we would assume that the release of HBV nuclear acids with EVs from hepatocytes will increase.

In HCV-infection, EVs containing viral RNAs may contribute to the transmission of HCV infection to healthy hepatocytes (Maji et al., 2017). Since HCV enhances exosome release from infected Huh 7.5.1 cells (Bukong et al., 2014), alcohol by increasing EV release in hepatocytes, will promote the spread of HCV-infection in people suffering from ALD. In fact, the release of large EVs, apoptotic bodies, from HCV-infected hepatocytes was enhanced by exposure to ethanol metabolites, and engulfment of these apoptotic bodies by macrophages induced inflammasome activation, while engulfment by HSC enhanced pro-fibrotic activation (Ganesan et al., 2016, 2018b).

## Alcohol and HIV-Induced Liver Disease

HIV is not a classic hepatotropic infection. However, ALT elevation is frequently observed in HIV-infected people (Mata-Marin et al., 2009). Also, some clinical studies addressed liver damage and progression to end-stage diseases in HIV patients (Debes et al., 2016; Sherman et al., 2017). Alcohol consumption is higher in people living with HIV (PLWH) than in general population (Bonacini, 2011) and may potentiate HIV-liver pathogenesis. Importantly, in HIV-infection in hepatocytes, alcohol/ethanol metabolites promoted apoptosis, which is similar to one observed in HCV-infection. These apoptotic bodies which contain HIV particles induce inflammasome activation in macrophages and profibrotic activation in HSC (Ganesan et al., 2019b). As reported in the same study, pro-fibrotic effects of apoptotic bodies were characterized as hepatocyte-specific because they were not triggered by apoptotic bodies generated from HIV-containing immune cells (Ganesan et al., 2019b). This explains our special interest to cell-to-cell communications in the liver in the setting of HIV-infection and alcohol exposure.

The progression to end-stage liver disease in alcohol abusers becomes even more rapid in people chronically infected with HIV. HIV infection impairs liver function, and despite improved survival due to antiretroviral therapy, liver-related disease in HIV-infected patients is one of the leading causes of non-AIDS-related death (Sherman et al., 2015; Dagur et al., 2018). Excessive alcohol consumption increases HIV viral load, suppresses the immune response, and promotes non-adherence to HIV treatment. Even low levels of alcohol use increase mortality in PLWH (Justice et al., 2016; Surial et al., 2021). Alcohol contributes to the accumulation of HIV proteins due to their impaired degradation in the liver by interfering with lysosomal biogenesis and activity, thereby leading to harmful hepatotoxic effects and potentiating HIV-mediated liver damage (Ganesan et al., 2019b; New-Aaron et al., 2021).

## Alcohol-and-HIV Induced EVs Contribution to Liver Disease Progression

Alcohol abuse exacerbates disease pathogenesis by causing oxidative stress, hepatotoxicity, and promoting HIV accumulation in HCs as well as EV-mediated transference of regulatory molecules from donor to recipient cells (Ganesan et al., 2019b; Rahman et al., 2020). Previous studies from the Osna laboratory demonstrated that the ethanol metabolite acetaldehyde, combined with HIV-infection, induced formation of apoptotic bodies from the HIV- and ethanol-exposed HCs, thereby providing detrimental consequences by activating non-parenchymal cells (Ganesan et al., 2019b). A recent study conducted on alcohol-fed humanized mice and alcohol-exposed human HCs demonstrated a reduced expression of lysosomal membrane protein (LAMP1), suppression of cathepsin B and L activities, and inhibition of cathepsin D enzyme maturation in the liver tissue with an increase in the secretion of human HC-derived EVs (Dagur et al., 2021).

**Figure 3:**
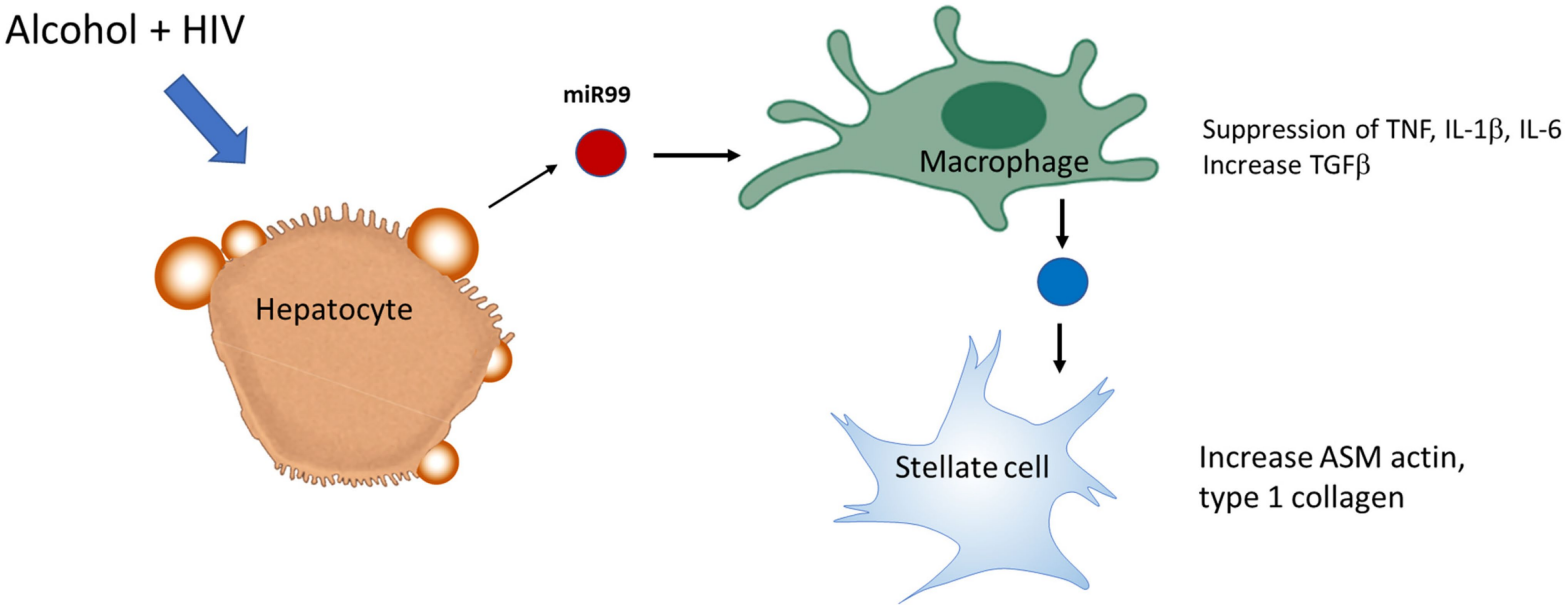
Extracellular vesicles derived from HIV-EtOH exposed hepatocytes (HCs) switch macrophage phenotype from M1 to M2 in HIV-infected macrophages. HCs activated by HIV and ethanol secrete EVs, which contain miRs (as an example, miR99). Engulfment of these exosomes by macrophages switches M1 macrophage phenotype to M2, thereby providing pro-fibrotic activation of hepatic stellate cell (HSC).

Circulating monocyte-derived macrophages (MDMs) infiltrate liver during organ injury and replenish the resident macrophage population (KCs) in homeostasis (van der Heide et al., 2019). A recent study assessed the effect of ethanol on HIV replication in MDMs and observed that chronic exposure of alcohol increased HIV replication by ~3-fold in HIV-1-infected MDM, which was associated with increased oxidative DNA damage, expression of CYP2E1, and a decreased expression of the antioxidant enzyme PRDX6 (Gong et al., 2019). *In vitro* polarization into M1 cells caused a marked decrease in the expression of HIV DNA, whereas in mature macrophages already infected with HIV, IL-4 stimulation enhances HIV-1 transcription without affecting the levels of CCR5 expression and showed no inhibition of viral DNA at an early stage (Cassol et al., 2010). Although the M1 phenotype marker TNFα is considered to be an activator of HIV-1 replication, TNFα has also been demonstrated to protect against HIV by stimulating the production of RANTES and decreasing CCR5 expression in macrophages (Lane et al., 1999). A recent study demonstrated that miR-99a expression is negatively correlated with inflammation by targeting TNFα, and it showed that overexpression of miR-99a prevented M1 phenotype activation promoting a phenotype switching to M2 (Jaiswal et al., 2019). Alcohol-HIV treatment of primary human HCs caused upregulation of four EV-miRNAs (miR-99a, miR-16, miR-122, and miR-17HG) and downregulated 10 miRNAs. KEGG pathway analysis demonstrated that metabolic pathways, such as the MAPK signaling pathway, ubiquitin-mediated proteolysis, the insulin signaling pathway, etc. were significantly upregulated by miRNAs in hepatocytes from HIV-alcohol group compared to either alcohol or HIV only groups. Mapping of miRNA target genes on KEGG disease database identified pathways associated with liver disease i.e., alcohol-related disorders, insulin resistance, fatty liver, fibrosis, inflammation in HIV-EtOH HIV treated groups. A maximum increase in the miR-99 containing EVs from HCs treated with alcohol-HIV was reported (Dagur, 2019). To summarize this part, exposure of miR-containing HC EVs to human macrophages, MDM and THP1 cells, switched macrophage phenotype from M1 to M2 and increased the level of HIV-infection in macrophages exposed to HIV (Figure 3).

## Crosstalk Between Cell Death and Inflammasome Activation in ALD

Extracellular vesicles and miRs as a part of EV cargo, have diverse functions in cell-to-cell communications including triggering liver inflammation/fibrosis. The pathogenesis of ALD is also characterized by the activation of inflammasome in the liver (Knorr et al., 2020). The inflammasome is a multiprotein complex comprising of a sensor (e.g., NLRP3, AIM2, and NLRC4), an adaptor protein (ASC), and an effector protein (caspase-1). Once stimulated, the sensor protein oligomerizes and recruits ASC, which then interacts with caspase-1 for proteolytic cleavage of pro-IL-1β and pro-IL-18, and their further release into the extracellular space (Broz and Dixit, 2016).

IL-1β, IL-18, and caspase-1 levels have been shown to be increased in the liver of ALD patients, and in a mouse model of ALD (Petrasek et al., 2012; Voican et al., 2015). The Szabo laboratory found that mice deficient in ASC or in caspase-1 have decreased hepatic and systemic IL-1β levels and showed protection from alcohol-induced liver damage, demonstrating the dependence on the adaptor protein ASC and caspase-1 on the increase of IL-1β in ALD. In the same work, it was also concluded that the primary source of IL-1β in ALD are macrophages/Kupffer cells. Analyses of liver mononuclear cells (LMNCs) isolated from mice treated with EtOH showed an increase in cleaved caspase-1 and IL-1β compared to saline treated mice. Furthermore, *in vivo* depletion of macrophages, followed by transplantation of caspase-1 KO bone marrow into WT mice, decreased alcohol-induced liver injury and hepatocyte damage (Petrasek et al., 2012), suggesting a major role of KCs in inflammasome activation in ALD.

**Figure 4:**
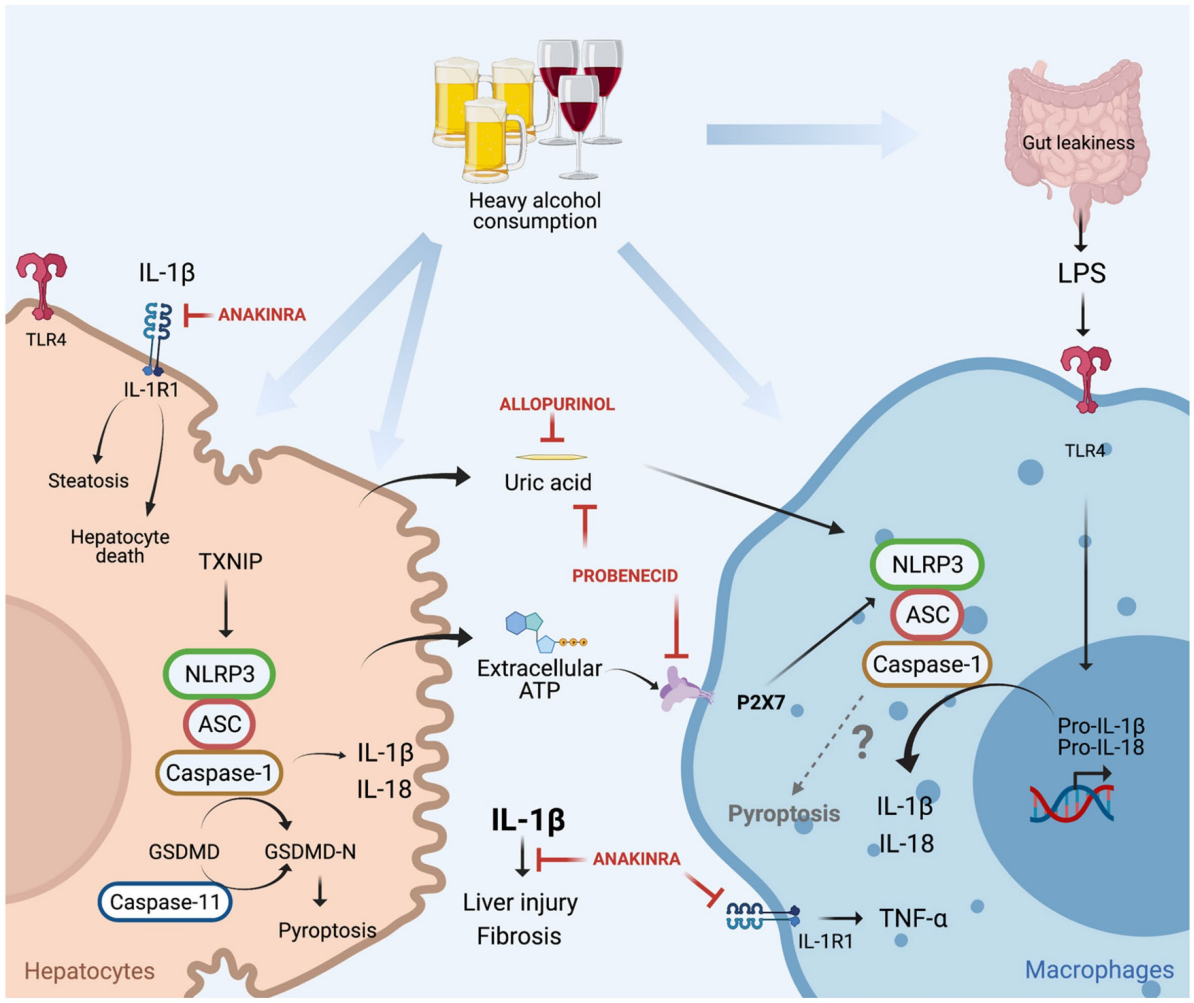
Canonical (NLRP3/caspase-1) and non-canonical (caspase-11) inflammasome induced cell death in alcoholic-associated liver disease. Heavy alcohol consumption induces hepatocyte death leading to the release of damage-associated molecular pattern (DAMPs; ATP and uric acid) into the circulation, in addition to the translocation of gut-derived LPS to the liver as a result of leaky gut. LPS through TLR4 induces pro-IL-1β and pro-IL-18 transcription in macrophages. Hepatocyte-derived extracellular ATP, through P2X7 signaling, and uric acid promote NLRP3 assembly, caspase-1 activation and cleavage of pro-IL-1β into bioactive IL-1β in LPS-stimulated macrophages for IL-1β release. IL-1β binds to IL-1R1 which induces hepatocytes triglycerides accumulation, hepatocyte death, macrophage activation, liver injury, and fibrosis. These effects are attenuated by treatment with anakinra (IL-1R1 antagonist), allopurinol (uric acid synthesis inhibitor), or probenecid (uric acid reducer and ATP signaling blocker). In hepatocytes, alcohol induces NLRP3/caspase-1 activation through TNXP1 protein interaction, culminating in pyroptosis. Alcohol-induced activation of caspase-11 in the liver, and overexpression of constitutively active gasdermin D (GSDMD) in hepatocytes leads to pyroptosis and liver damage.

IL-1β, mainly produced by hepatic macrophages, binds to IL-1R1, and promotes the accumulation of triglycerides in HCs, induces HCs cell death, immune cells activation and recruitment to the liver (invariant natural killer T cells and neutrophils) as well as the development of fibrosis (Petrasek et al., 2011, 2012; Cui et al., 2015). Mice deficient in IL-1R1 (IL-1R1 KO) presented less liver injury compared to WT mice under chronic alcohol feeding. In addition, treatment with Anakinra (IL-1R1 antagonist) decreased liver damage in chronic alcohol-fed WT mice and accelerated liver regeneration in a mouse model of ethanol-induced acute-on-chronic liver injury (Petrasek et al., 2011, 2012; Iracheta-Vellve et al., 2017; Figure 4).

In another study, NLRP3 was identified as the sensor protein involved in inflammasome activation in ALD, as NLRP3-deficient mice had less alcohol-induced liver damage compared to alcohol-treated WT mice (Petrasek et al., 2015). The signals that induced NLRP3 inflammasome activation and the interaction between the cells that produce and receive those signals have also been reported in ALD. NLRP3 inflammasome is commonly activated in a two-step process, where the first step is characterized by transcription and upregulation of inflammasome components (pro-IL-1β, pro-IL-18, and NLRP3) mediated by NFκB. The second step of inflammasome activation culminates in the assembly of the NLRP3 complex for caspase-1 activation and IL-1β release, commonly mediated by damage-associated molecular patterns (DAMPs; Broz and Dixit, 2016). It has been demonstrated that gut derived PAMPs, such as LPS are increased in the serum of ALD patients and in mouse models of ALD and induce upregulation of inflammasome components through TLR4/NFkB activation in macrophages. In addition, healthy volunteers exposed to a single dose of ethanol had increased circulating levels of endogenous DAMPs: ATP and uric acid. The origin of these molecules is likely damaged HCs, as *in vitro* alcohol exposure to HCs induces cell death in addition to an increase in the release of uric acid and ATP (Petrasek et al., 2015). These hepatocytes derived DAMPs activate the NLRP3 inflammasome in LPS-exposed liver mononuclear cells by IL-1β release, indicating that ATP and uric acid function as second signals for macrophages inflammasome activation in ALD.

The importance of ATP and uric acid in the activation of NLRP3 in ALD was additionally explored by the Szabo laboratory in different mouse models (Iracheta-Vellve et al., 2015). Firstly, mice that lack ATP signaling through P2X purinoceptor 7 (P2X7 KO mice), presented less inflammasome activation in the liver and decreased levels of IL-1β in the serum, in addition to less HC damage. Secondly, the role of uric acid in inflammasome activation in ALD was unraveled in mice that overexpress uricase, an enzyme that depletes uric acid. Similar conclusions were drawn using a pharmacological approach, where mice were treated with allopurinol or probenecid. The former prevents uric acid synthesis, and the latter induces uric acid depletion and blocks ATP signaling. Both the genetic and pharmacological approaches for decreasing uric acid availability in ALD decreased ethanol-induced liver injury (Iracheta-Vellve et al., 2015; Figure 4). Furthermore, it has been reported that in ALD, EVs as a second signal for inflammasome activation. Momen-Heravi et al. (2015) demonstrated that EVs, derived from alcohol-stimulated hepatocytes, promote the release of IL-1β from LPS-stimulated monocytes.

In addition to IL-1β release, the activation of the NLRP3 inflammasome also induces a cell death called pyroptosis through caspase-1 mediated cleavage of gasdermin D (GSDMD). Cleaved GSDMD binds to the membrane and forms pores, responsible initially for IL-1β and IL-18 secretion and later for pyroptosis (Swanson et al., 2019). Heo et al. (2019) demonstrated *in vitro* that EtOH-treated HCs promote caspase-1 activation, IL-1β release and pyroptosis, through the binding of the thioredoxin-interacting protein (TXNIP) to NLRP3 (Figure 4). This finding supports *in vivo* data in mice overexpressing a constitutively active global NLRP3, which increases liver damage caused by HC pyroptosis and liver inflammation, and HSC activation resulting in fibrosis (Wree et al., 2014). Khanova et al. (2018) demonstrated that in AH, pyroptosis is mediated by a non-canonical inflammasome through caspase-11 activation, as caspase-11 KO mice decrease GSDMD activation in the liver and are protected from AH, induced by an intragastric chronic alcohol feeding plus *ad libitum* western diet together with weekly alcohol binge. In addition, the *in vivo* overexpression of a constitutively active GSDMD in HCs, showed increased HC death in mice under intragastric alcohol feeding (Khanova et al., 2018). These works notably demonstrated that in ALD, pyroptosis in hepatocytes could be mediated by caspase-11 or NLRP3/caspase-1 activation, which contribute for liver damage and fibrosis. However, the role of pyroptosis in immune cells in ALD needs detailed investigation and the participation of NLRP3 inflammasome in activating pyroptosis in immune cells in ALD is yet to be understood.

Upon NLRP3 inflammasome activation, the adaptor protein ASC oligomerizes and forms a macromolecular structure named ASC specks. It has been shown that in macrophages that undergo pyroptosis mediated by NLRP3, ASC specks are released (Franklin et al., 2014). Our further studies will explore whether the activation of NLRP3 observed in ALD leads to the release of pyroptosis-mediated ASC specks, and will explore these phenomena in the possible interaction between parenchymal and immune cells contributing to the pathogenesis of ALD.

## Role of Sinusoidal Pressure and Stellate Cell Stretch Forces in ALD

In addition to EV exchange or inflammation, “biomechanical” factors, like sinusoidal pressure and hepatic arterilization promote fibrosis/cirrhosis development even in the absence of inflammation. These “biomechanical” triggers and can be included in the list of the factors, which affect cell–cell communication leading to pathomorphological end-stage changes in the liver.

## Established Mechanisms of Liver Cirrhosis

Chronic liver diseases, including heavy alcohol consumption, frequently lead to scarring (cirrhosis), a process in which the architectural organization of functional liver units becomes disrupted. Liver cirrhosis is the result of excessive accumulation of extracellular matrix (ECM). This is often accompanied by progressive loss of organ function despite the use of immunosuppressive, anti-viral or anti-inflammatory agents (Bataller and Brenner, 2005; Schuppan and Afdhal, 2008). In the Western world, many patients with liver cirrhosis die from hepatocellular carcinoma (HCC), which is the sixth most common cancer worldwide. In addition, HCC ranks at the third place in cancer-related mortality statistics and shows the second fastest growth rate (Lozano et al., 2012; Fares and Peron, 2013). More than 90% of HCCs develop in cirrhotic livers mostly due to ALD or chronic viral hepatitis (HBV or HCV; Berres et al., 2009; Seitz and Mueller, 2009). Currently, the progression of fibrosis to cirrhosis is most efficiently blocked by treating the underlying disease and no satisfying anti-fibrotic treatment regimens exist to directly attack fibrotic processes.

**Figure 5:**
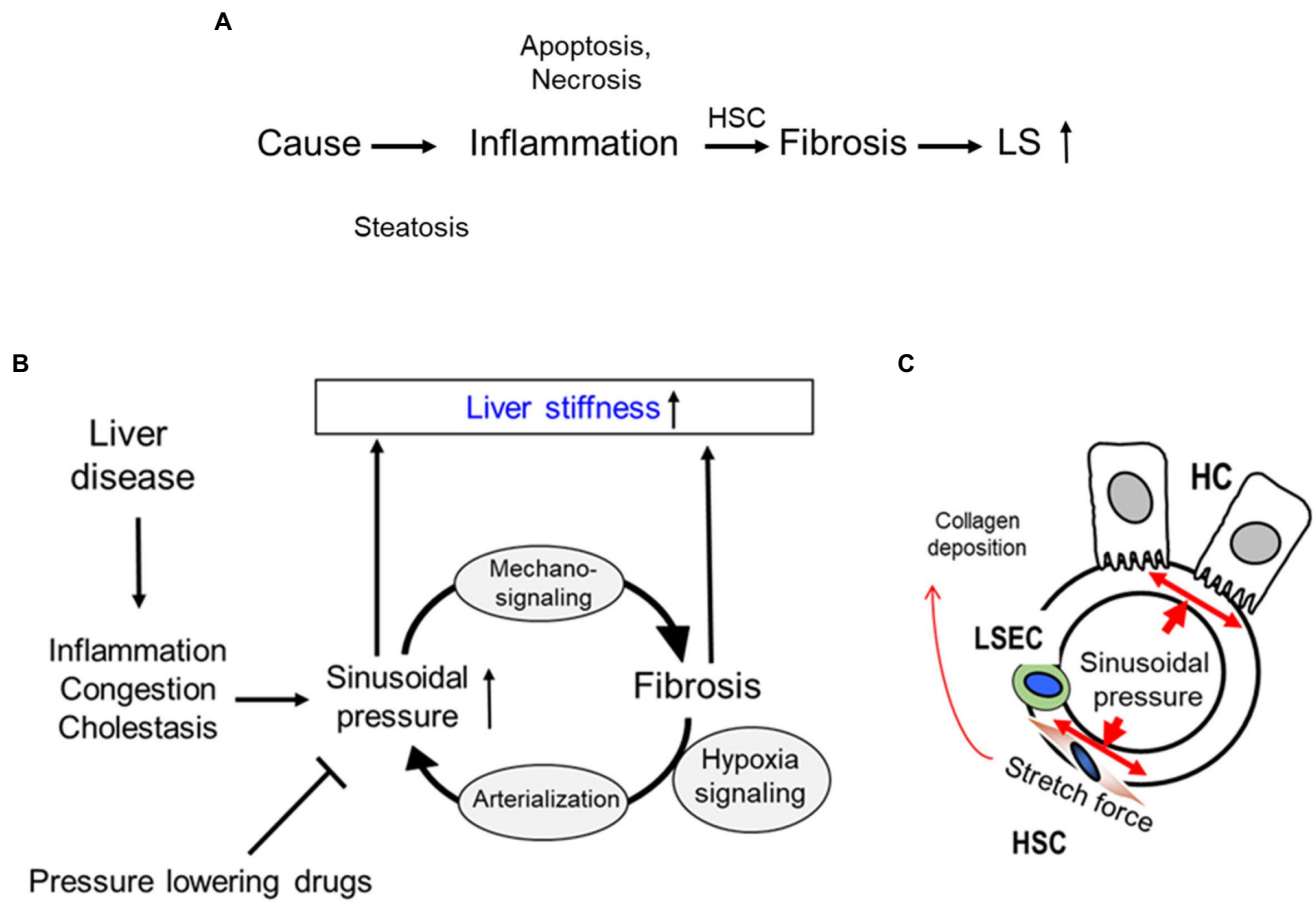
Sinusoidial pressure hypothesis (SPH) at the organ and cellular level. **(A)** Conventional sequence of events during fibrosis progression with elevated liver stiffness (LS) mostly seen as a final consequence of matrix deposition. HSC, hepatic stellate cells; LS, liver stiffness. **(B)** All liver pathologies cause sinusoidal pressure (SP) elevation that ultimately drives fibrosis through mechano-signaling. A sustained pressure elevation ultimately causes arterialization of the liver *via* hypoxia signaling. SPH identifies arterialization as point of no return for liver progression and is able to explain macroscopic changes. Moreover, within the concept of SPH, pressure-lowering drugs could not only be used to treat complications of cirrhosis such as portal hypertension but causally to interrupt the vicious cycle by lowering the sinusoidal pressure. **(C)** According to SPH, SP predominantly translates into mechanic stretch forces within the perisinusoidal bed. Hepatocyte cell death, inflammation, or congestion all lead to increased SP that causes stretching of e.g., HSC, liver sinus endothelial cells (LSEC), or HC.

The mechanisms of hepatic fibrosis are complex and not fully understood. A variety of adverse stimuli, such as hepatotoxins, viruses, bile acids, and hypoxia can trigger fibrogenesis and so-called reactive oxygen species seem to play an important role in fibrosis progression (Benyon and Arthur, 2001; Schuppan et al., 2001, 2003). Major proteins of the ECM are collagens forming important scaffolds and barriers. In addition, proteolysis of collagens by specific proteases appears to be rate limiting for ECM removal. Collagen type I, III, and IV are the most abundant ECM components in the liver and their relative content increases up to 10-fold in cirrhosis (Schuppan, 1998; Wells, 2013). In the acute phase of liver disease, fibrosis is a dynamic process, in which fibrogenesis is usually counterbalanced by fibrolysis, i.e., the removal of excess ECM by proteolytic enzymes, most importantly by matrix metalloproteinases (MMPs). With repeated injury or sufficient severity, fibrogenesis prevails over fibrolysis, resulting in excess ECM synthesis and deposition, a downregulation of MMP synthesis, secretion and activity along with an increase of the tissue inhibitors of MMPs (TIMPs, especially TIMP-1). ECM components, MMPs, and TIMPs are mainly produced by activated HSCs and fibroblasts (Olaso and Friedman, 1998). Activated KCs and also other cells are a major source for fibrogenic cytokines, such as TGF-β known as the master cytokine of fibrosis development, which further stimulates HSC and fibroblasts to transdifferentiate into activated myofibroblasts. Myofibroblasts are the main cell type responsible for excess matrix deposition at the sites of tissue repair (Weiskirchen, 2015). According to the concept presented by Weiskirchen (2015), liver becomes stiffer due to increased matrix deposition and therefore, results in elevated liver stiffness (LS) that is the final consequence of liver fibrosis (Figure 5A).

## Introducing the Role of Pressure in Driving Fibrosis Progression: The Sinusoidal Pressure Hypothesis

The potential role of sinusoidal pressure in mediating fibrosis progression has been recently recognized mainly through the novel *in vivo* data on transient elastography (TE) obtained from both clinical and animal studies (Mueller and Sandrin, 2010; Mueller, 2016). TE has significantly improved the non-invasive assessment and screening for liver cirrhosis (Sandrin et al., 2003; Mueller and Sandrin, 2010) and also led to generating novel mechanistic concepts. During fibrosis progression, LS increases continuously from 2 kPa up to 75 kPa. A threshold of 12.5 kPa is now widely considered as cut-off value of histological F4 cirrhosis stage (Friedrich-Rust et al., 2008). However, many laboratories recognized previously that various other conditions are able to elevate LS irrespective of fibrosis. These conditions include liver inflammation (hepatitis; Arena et al., 2008; Sagir et al., 2008), liver congestion (Millonig et al., 2010), and mechanic obstruction of bile ducts (cholestasis; Millonig et al., 2008). The observation that the central venous pressure as well as the intra-ductal biliary pressure are able to reversibly increase LS without any other confounders, such as inflammation. (Millonig et al., 2008, 2010) suggested an important role of the sinusoidal pressure as an etiological factor of fibrosis progression, which led to postulate the sinusoidal pressure hypothesis (SPH; Millonig et al., 2010; Mueller and Sandrin, 2010; Mueller, 2020e). In addition to the findings mentioned above, well established data from more than 500 clinical studies are most relevant to the concept of SPH (Mueller et al., 2010; Mueller, 2020a,b,c,d; Mueller and Lackner, 2020; Rausch et al., 2020). SPH provides an answer to all these open questions based on biomechanic considerations (Mueller, 2016). Figure 5B summarizes the SPH as follows: All potential causes of cirrhosis ultimately lead to an elevated SP. SP consists of dynamic and static components, such as hepatic inflow and outflow balances or water retention. Even minimal increases of SP seem to be critical for the low-pressure organ liver, which is typically exposed to *ca*. 5 mmHg *via* the portal vein. This also means that LS almost exclusively mirrors SP in the absence of fibrosis. At the cellular level (Figure 5C), SP is the actual driving force for the production of ECM by stretching of peri-sinusoidal cells e.g., HSCs, fibroblasts, and LSECs. It is still unclear whether these cells simply “feel” the surrounding pressure-mediated stiffness by dedicated sensing mechanisms (Discher et al., 2005) or whether they directly sense pressure-mediated stretch forces. So far, stiffness-mediated activation of HSC has not been linked to pressure or SP (Hinz, 2009; Wells, 2013). According to the physics of mechanics, it is easily conceivable that pressure-induced stretch forces will overlay at the whole organ levels leading to regions with high trajectory forces and consequent large septa formation. SP-mediated stretch forces and matrix are in continuous equilibrium. Dosage and time of elevated SP/LS determine fibrosis progression (biomechanical signaling) eventually leading to a degree of matrix deposition that “matches” the pressure. Experimental and common clinical observations suggest that a SP > 10–12 mmHg or a LS > 10–12 kPa and period of time > 4–6 weeks are critical thresholds.

## Fibrosis Perpetuation by Arterialization of the Fibrotic Liver and Continuous Pressure Elevation

The hepatic artery is directly connected to the sinusoidal bed *via* arteriole inlets and provides about 20% of blood in a normal healthy liver. When liver becomes stiffer due to inflammation or fibrosis, more pressure is required to maintain a sufficient blood flow. Although the elevation of portal pressure (portal hypertension > 12 mmHg) can partly maintain some portal flow, it will hardly reach values higher than 30 mmHg. Under these conditions, the hepatic artery will be the only vessel with sufficiently high pressure to maintain hepatic blood supply. Elevation of hepatic arterial flow and subsequent arterialization is mainly driven by hypoxia signaling (Medina et al., 2004). SPH postulates that this arterialization defines the so-called “point of no return.” It provides a pressure-based rationale to explain the self-perpetuation of fibrosis progression and the uniform, etiology-independent progression of fibrosis. Arterialization of the fibrotic liver ultimately leads to a sustained exposure of the low-pressure organ liver (typically <6 mmHg) to higher pressures (see also Figure 5). Current investigations are underway in experimental animal models to examine whether sustained pressure elevation can cause significant liver fibrosis even in the absence of inflammation and whether increasing stiffness requires more arterial supply. In support of this concept, a large multicenter trial demonstrated that LS values are indeed higher in lobular liver diseases, such as ALD as compared to portal HCV (Mueller et al., 2015). Moreover, it was also shown that patients with HCV infection have higher spleen stiffness and portal pressure than patients with ALD, within the same fibrosis stage, which is matched to LS. Additionally, patients with HCV more commonly progressed to portal hypertension-related complications (e.g., variceal bleeding), while patients with ALD more commonly progress to liver failure (e.g., jaundice; Elshaarawy et al., 2019). Thus, biomechanical aspects of liver diseases, such as pressure and the resulting stiffness deserve further consideration to better understand the underlying molecular mechanisms of ALD.

Importantly, an increased LS correlates with a rise in HCV- and HIV RNA levels in hepatocytes co-infected with HCV and HIV to promote apoptosis of virally infected cells (Ganesan et al., 2018a), which upon uptake by HSCs induce their activation and aberrant ECM production. Thus, LS serves as an important factor contributing to further cirrhosis progression in people co-infected with HCV and HIV.

## Multifactorial Regulation of Cell-to-Cell Communications

As mentioned in Introduction, the factors regulating cell-to-cell communication may work interdependently (by affecting each other) or separately. The interactions between EVs and inflammasome have been already reported in several review articles (Cannito et al., 2017; Cypryk et al., 2018; Noonin and Thongboonkerd, 2021). As stated in these reviews, there is strong evidence that inflammasome activity correlates with EV release and due to common activators of inflammasome and exosome release, inflammasome was considered as an enhancer for EVs secretion; however, not all studies support this. Furthermore, EVs released from inflammasome-activated macrophages carry a specific RNA signature and contain interferon β (Budden et al., 2021), which is important for anti-viral cell protection. Most of these publications are not related to ALD, with few exclusions. Thus, as reported, in ALD, EVs serve as a second signal for inflammasome activation, and Momen-Heravi et al. (2015) demonstrated that EVs, derived from alcohol-stimulated hepatocytes promote the release of IL-1β from LPS-stimulated monocytes. In addition, exosomes derived from alcohol-stimulated hepatocytes contain mitochondrial double-stranded RNA and trigger IL-1β release from Kupffer cells (Lee et al., 2020). Also, large apoptotic bodies derived from ethanol-exposed hepatocytes activate inflammasome after engulfment by macrophages (Ganesan et al., 2019b).

The interactions between liver stiffness and EV release and inflammasome have not been published yet. However, in experimental settings, hepatocytes plated on the stiff surface (corresponding to stiffness at liver cirrhosis) and co-cultured with fibroblasts have decreased albumin synthesis and cytochrome P450 activity vs. the same cells plated on the surface with normal stiffness (Natarajan et al., 2021). We also observed increased release of EVs from hepatocytes exposed to ethanol and plated on stiff surface (Osna’s laboratory, unpublished observations). The mechanisms behind this event are still under debates.

Overall, currently, the complex interactions between EVs, inflammasome, and liver stiffness in ALD are not fully established and require more detailed clarification.

## Conclusion

In this review, we have highlighted two fundamental types of cell–cell communication phenomena that lead to the development of advanced ALD. Thus, molecular signal molecules released from one cell type affect other cell types in the liver. This has been shown for liver cell-to cell communications *via* EVs that play a role in promoting end-stage liver disease development. This information exchange and programming of cell recipient cell functions depends on EVs secreted by donor liver cells in response to alcohol, is even more robust in virally infected cells, and can be attributed to specific miRs, mtDNA, and proteins in the EVs cargo. Alcohol-mediated inflammasome activation in parenchymal and non-parenchymal liver cells induces cell death *via* pyroptosis thereby affecting disease sates as well. In addition to the importance of molecular pro-inflammatory signals, a second type of communication signal can be transmitted *via* sinusoidal pressure biomechanical factors that operate even in the absence of inflammation. These factors include an increased venous pressure and LS associated with a pronounced profibrogenic response and histological fibrosis progression in the absence of inflammation. Pressure and mechanic stimulation of HSC could be an important hitherto underestimated factor in the development of aberrant extracellular matrix deposition.

## Author Contributions

All authors listed have made a substantial, direct, and intellectual contribution to the work, and approved it for publication. All authors contributed equally for preparation of this manuscript.

## Funding

This work was supported, in whole or in part, by the United States Department of Veterans Affairs Biomedical Laboratory Research and Development Merit Review grants, BX004053 (KK), NIH grants R01AA026723 (KK), R01AA027189 (NO), K01AA026864 (MG), R01AA01286 (SAW), R01AA028134 (AF, HT), R01AA011567 (GS), R01AA17729 (GS), P60AA011999 (HT), R24AA012885 (HT), I01BX001991 (HT), and IK6BX004205 (HT).

## Conflict of Interest

GS reports being a paid consultant for Evive Bio, Merck, Novartis, Durect Corporation, Terra Firma, Cyto Therapeutics, Pfizer, Surrozen; she holds stock in Glympse, Zomagen Biosciene/Ventyx Biosciences and receives royalties from Springer Nature Group and UpToDate Inc. All other authors reported no conflict of interests.

## Publisher’s Note

All claims expressed in this article are solely those of the authors and do not necessarily represent those of their affiliated organizations, or those of the publisher, the editors and the reviewers. Any product that may be evaluated in this article, or claim that may be made by its manufacturer, is not guaranteed or endorsed by the publisher.
